# Supplementary material for: Analysis of partial sequences of the RNA-dependent RNA polymerase gene as a tool for genus and subgenus classification of coronaviruses
Source: J Gen Virol. 2020 Sep 9;101(12):1261–9. doi: 10.1099/jgv.0.001494 (PMC7819353; doi:10.1099/jgv.0.001494)
Supplement: Supplementary material 1 [file jgv-101-1261-s001.pdf]

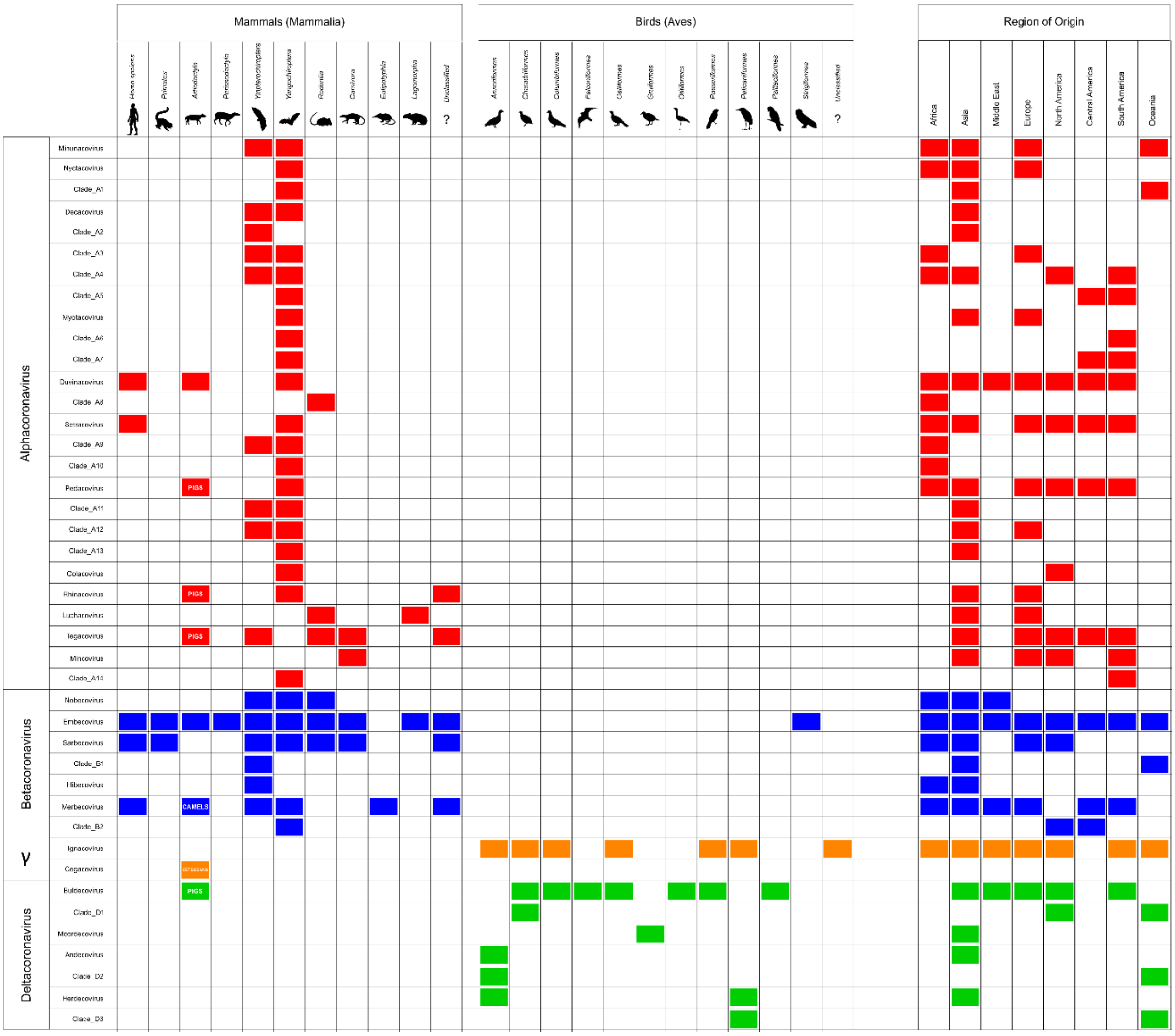

Supplementary Figure S1: Metadata (host and geographical origin) associations of Coronaviruses belonging to different official subgenera, and other unclassified major clade groups as depicted in main figures 2 and 3. Blocks of colour represent the existence of at least one record of the indicated association, and are coloured by viral genus as in main figure 1.

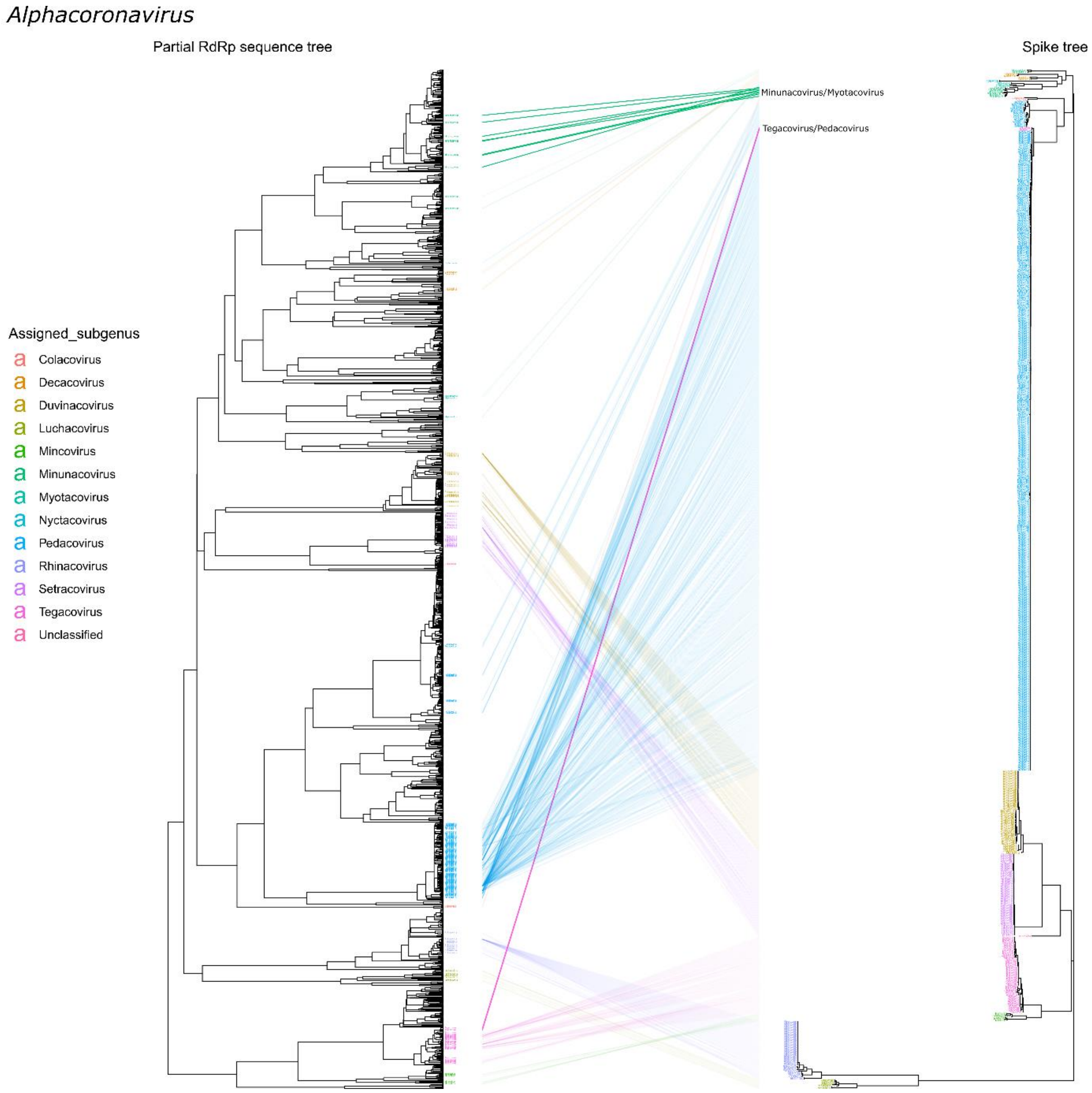

Supplementary Figure S2: Tanglegram representation of phylogenetic topologies estimated for Alphacoronaviruses using partial RdRp sequence data (left) and the spike gene sequence data (right). Correspondence between isolates is highlighted by lines. Colours indicate predicted subgenus of each isolate based on the analysis of the RdRp sequence. Lines in bold highlight sequences that would be classified differently at the level of subgenus when using the different genomic loci, and the differential classifications are labelled to the right of the highlighted lines.

Betacoronavirus

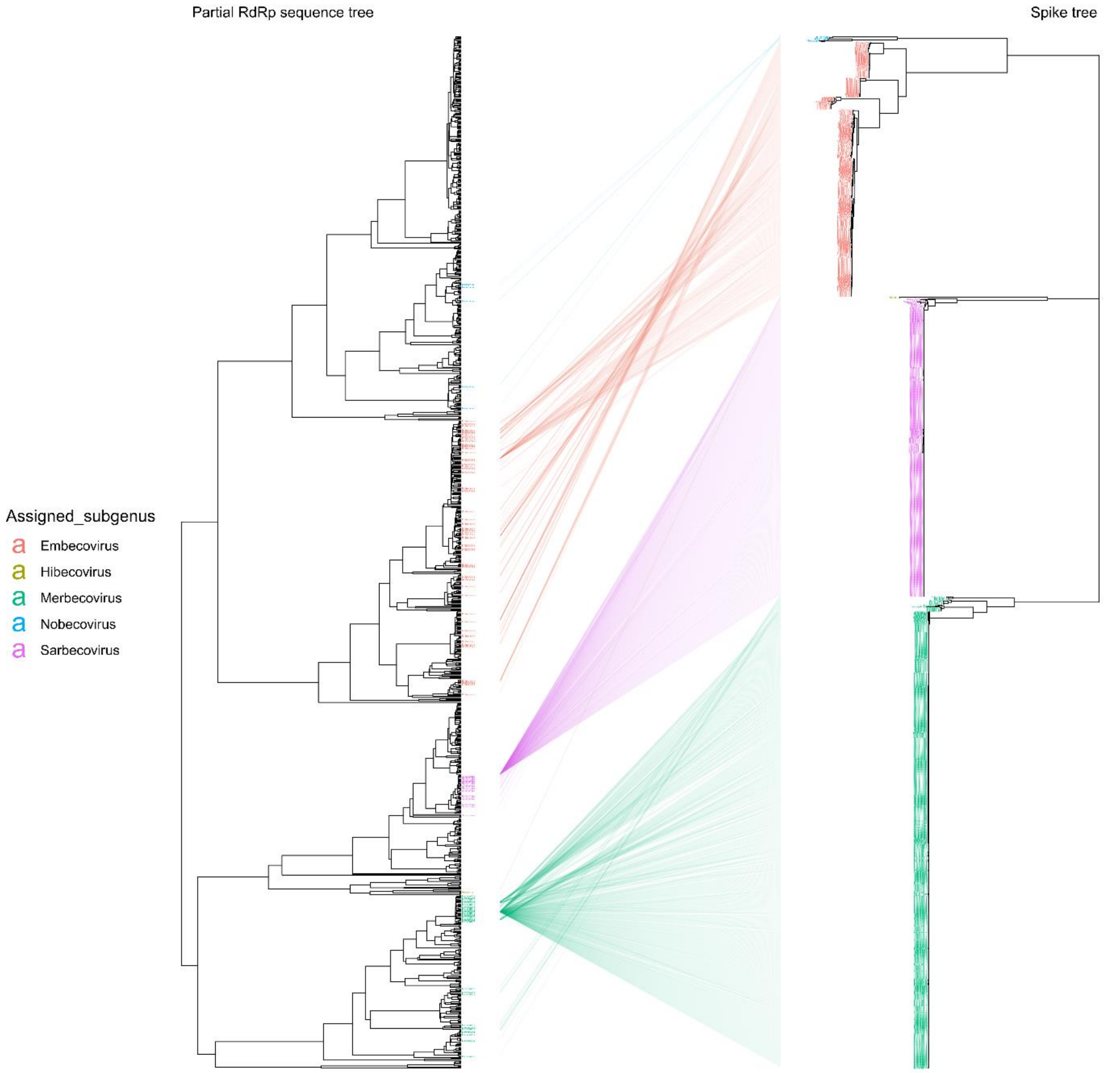

Supplementary Figure S3: Tanglegram representation of phylogenetic topologies estimated for Betacoronaviruses using partial RdRp sequence data (left) and the spike gene sequence data (right). Correspondence between isolates is highlighted by lines. Colours indicate predicted subgenus of each isolate based on the analysis of the RdRp sequence.

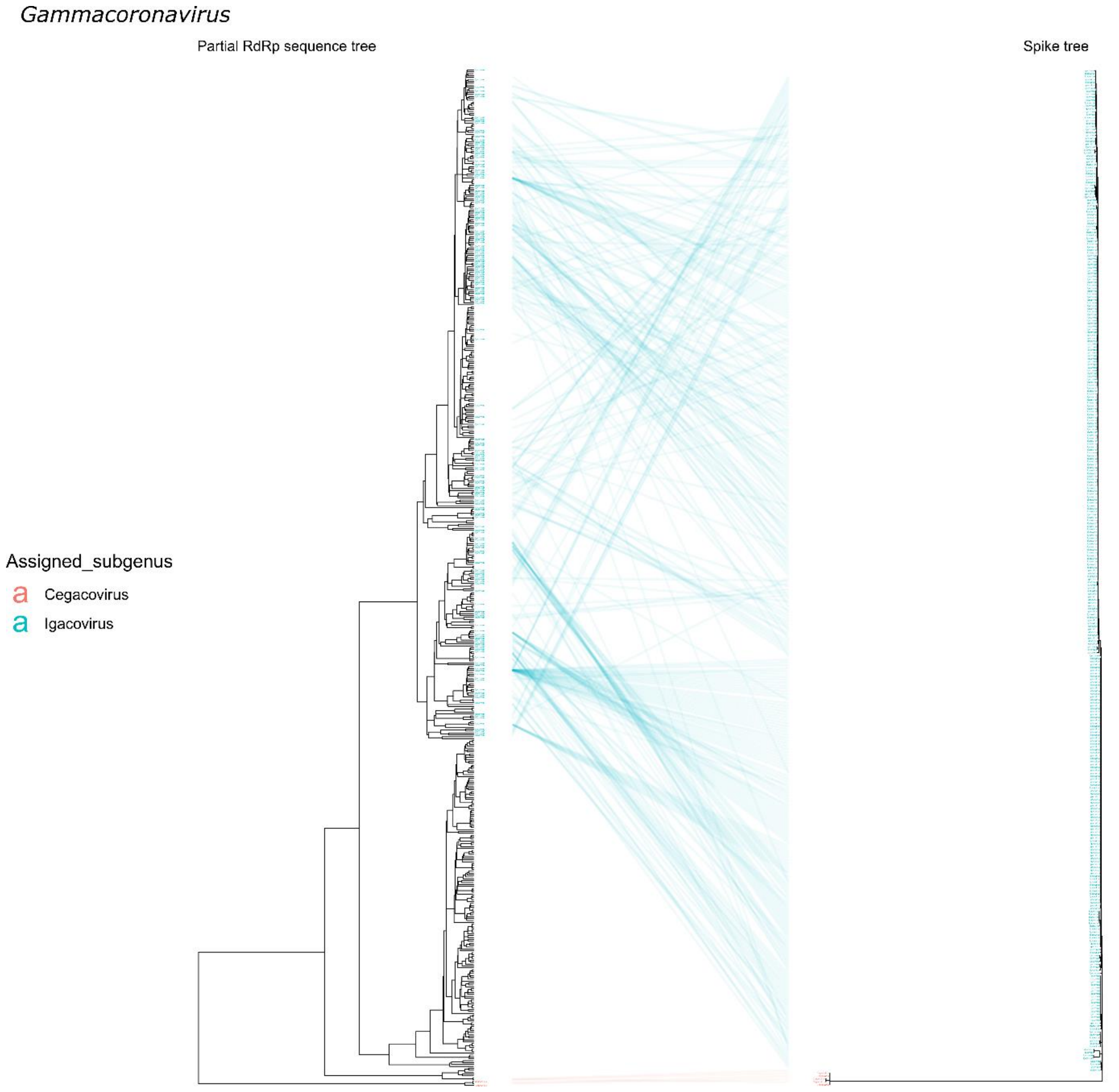

Supplementary Figure S4: Tanglegram representation of phylogenetic topologies estimated for Gammacoronaviruses using partial RdRp sequence data (left) and the spike gene sequence data (right). Correspondence between isolates is highlighted by lines. Colours indicate predicted subgenus of each isolate based on the analysis of the RdRp sequence.

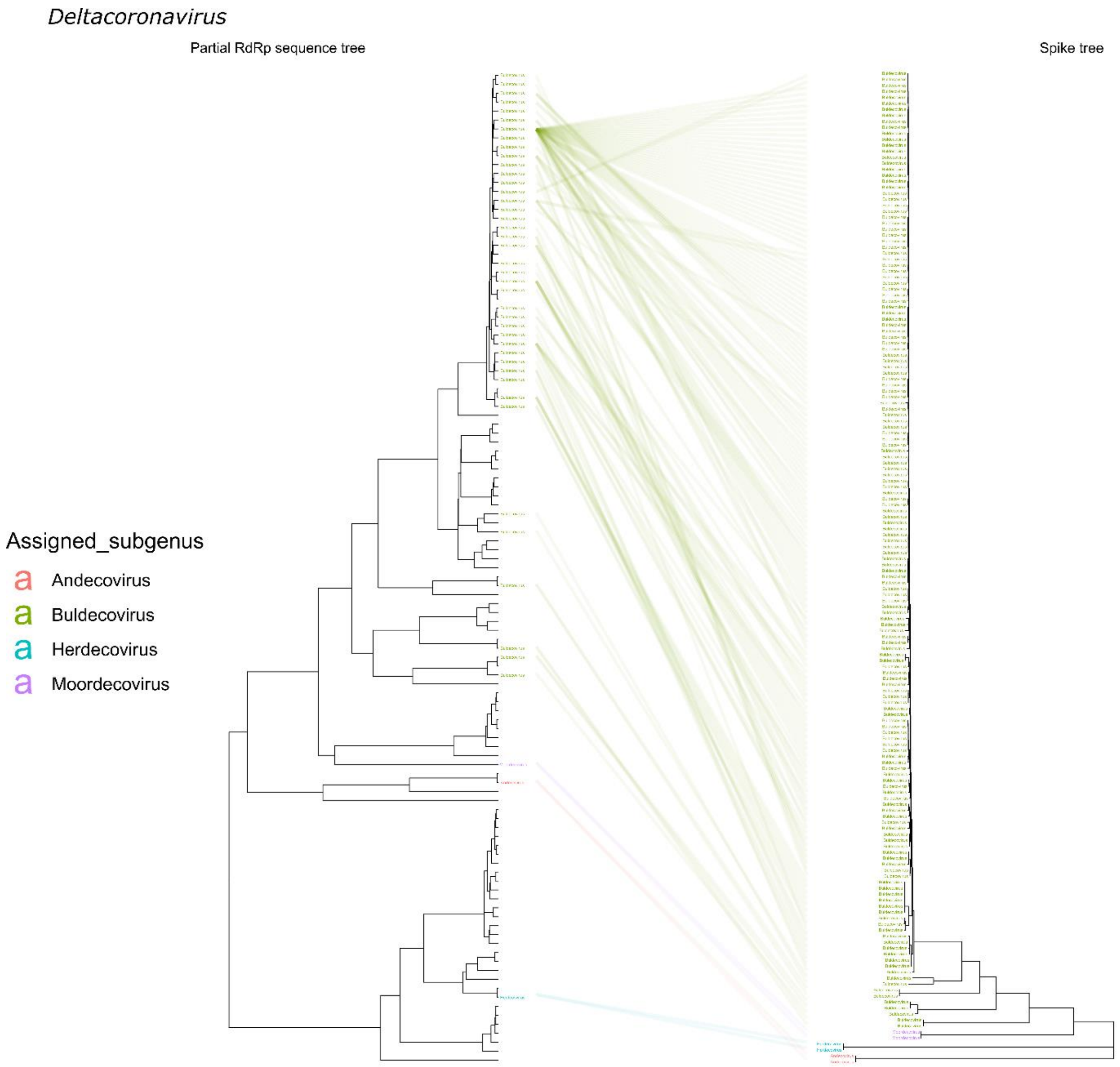

Supplementary Figure S5: Tanglegram representation of phylogenetic topologies estimated for Deltacoronaviruses using partial RdRp sequence data (left) and the spike gene sequence data (right). Correspondence between isolates is highlighted by lines. Colours indicate predicted subgenus of each isolate based on the analysis of the RdRp sequence.
